# Genomic-Scale Interaction Involving Complementary Sequences in the Hepatitis C Virus 5′UTR Domain IIa and the RNA-Dependent RNA Polymerase Coding Region Promotes Efficient Virus Replication

**DOI:** 10.3390/v11010017

**Published:** 2018-12-28

**Authors:** Elodie Rance, Jerome E. Tanner, Caroline Alfieri

**Affiliations:** 1 Laboratory of viral pathogenesis, Research Center, CHU Sainte-Justine, 3175 Côte Sainte-Catherine Road, Montréal, Québec H3T 1C5, Canada; elodie.rance@gmail.ca (E.R.); jetanner@videotron.ca (J.E.T.); 2 Département de microbiologie, infectiologie, immunologie, Université de Montréal, Québec, H3T 1C5, Canada

**Keywords:** NS5B, secondary structure, circular RNA, RNA folding, RNA stem-loop, HCV IRES, long-distance RNA–RNA interaction, *Flaviviridae*

## Abstract

The hepatitis C virus (HCV) genome contains structured elements thought to play important regulatory roles in viral RNA translation and replication processes. We used in vitro RNA binding assays to map interactions involving the HCV 5′UTR and distal sequences in NS5B to examine their impact on viral RNA replication. The data revealed that 5′UTR nucleotides (nt) 95–110 in the internal ribosome entry site (IRES) domain IIa and matching nt sequence 8528–8543 located in the RNA-dependent RNA polymerase coding region NS5B, form a high-affinity RNA-RNA complex in vitro. This duplex is composed of both wobble and Watson-Crick base-pairings, with the latter shown to be essential to the formation of the high-affinity duplex. HCV genomic RNA constructs containing mutations in domain IIa nt 95–110 or within the genomic RNA location comprising nt 8528–8543 displayed, on average, 5-fold less intracellular HCV RNA and 6-fold less infectious progeny virus. HCV genomic constructs containing complementary mutations for IRES domain IIa nt 95–110 and NS5B nt 8528–8543 restored intracellular HCV RNA and progeny virus titers to levels obtained for parental virus RNA. We conclude that this long-range duplex interaction between the IRES domain IIa and NS5B nt 8528–8543 is essential for optimal virus replication.

## 1. Introduction

Hepatitis C virus (HCV) is a hepatotropic RNA virus of the genus hepacivirus in the *Flaviviridae* family [1]. Members of this family have a single-stranded RNA genome of positive polarity containing a single long open reading frame (ORF) flanked by highly structured 5′ and 3′ untranslated regions (UTRs). Following HCV entry into the host cell and cytoplasmic release of the 9.6 kb HCV RNA genome, the 5′UTR internal ribosomal entry site (IRES) initiates polyprotein synthesis from the large ORF that is cleaved co- and post-translationally into three virion structural proteins and seven nonstructural (NS) replication-essential proteins [2]. Virus RNA replication proceeds upon assembly of NS and host cofactor proteins into vesicle-bound replication complexes for synthesis of a genomic-length negative (−)strand RNA intermediate [3]. The (−)strand RNA intermediate now serves as the amplification template for virion progeny RNAs.

Like the RNA genomes of other flaviviruses, HCV usurps the cell’s normal mRNA structural signals by forming local or distal genomic linkages that allow the virus to replicate through initial translation of the viral positive (+)strand, followed by synthesis of the (−)strand intermediate and, lastly, by synthesis and packaging of the progeny virus (+)strand RNA genome [4,5,6]. Structural and locational conservation of these structural elements in all seven known HCV genotypes, as well as experimental findings wherein genetic manipulations that disrupt genomic RNA structural integrity result in severe or complete disruption of progeny virus production, lend credence to the notion that conserved RNA secondary structures act as critically important guideposts or temporal controls during flavivirus replication [7,8].

The HCV 5′UTR is highly conserved and is predicted to fold into four high-order domain structures comprised of extended stem-loop (SL) structures interspersed with internal bulges or intra-domain loop junctions and a pseudoknot. While the principal function of the 5′UTR is to initiate viral RNA translation, there is also strong evidence indicating that the 5′UTR participates in and is essential for positioning the virus replication complex on the 3′UTR to begin (−)strand synthesis [9,10,11]. Consistent with the replication process of other members of the *Flaviviridae*, it is hypothesized that 5′UTR:NS5B-3′UTR RNA-RNA linkage and resulting genome circularization is an important first step for efficient virus RNA replication [12,13].

In addition to the conserved secondary structures in the UTR termini, a concentration of conserved SL structures initially mapped to the 3′-end of the viral RNA dependent RNA polymerase coding sequence, NS5B [5], have been shown to act as cis elements for the initiation of RNA (−)strand synthesis at the 3′UTR terminus [14,15]. Moreover, recent studies to catalogue additional genomic duplexes involved in (−)strand template synthesis using in vitro-folded genomic RNA followed by selective 2′-hydroxyl acylation analyzed by primer extension, SHARP [5,16], and genetic manipulation [17] have expanded the number and extent of duplex elements involved in HCV RNA synthesis to the 5′-end of NS5B. Knowledge regarding these additional RNA SL structures and their individual contribution to virus replication remains largely incomplete.

From in silico analysis [18], we hypothesized that a highly conserved yet uncharacterized RNA hetero-duplex formed by 5′UTR domain IIa nucleotides (nt) 95–110 and NS5B nt 8528–8543 may contribute to genome circularization and promote efficient HCV replication. Our findings using in vitro binding assays and analysis of virus RNA levels and HCV titers for viral mutants indicate that the RNA duplex comprised of these two distal genome sequences is required for optimal HCV RNA and progeny virus production.

## 2. Materials and Methods

### 2.1. Cell Culture

The human hepatoma cell line Huh-7.5 (kindly provided by Dr. Tatsuo Takahashi, Health Science Research Resources Bank, Japan) was cultured in Dulbecco’s Modified Eagle’s Medium (DMEM) supplemented with 10% fetal bovine serum and antibiotics (Wisent Bioproducts, St Bruno, QC, Canada).

### 2.2. Construction of HCV 1b Mutant Virus

The HCV genotype 1b RNA expression plasmid pER-1b was constructed from pGEM-7Zf-HCV1b plasmid and the addition of the 5′UTR microRNA-122 complementary sequence and HCV G1b 3′UTR [19,20]. Additional changes were introduced into pGEM T7 RNA polymerase promoter to restrict RNA transcription to the authentic HCV genome terminal guanine nucleotide (Table S1). Construction of virus mutants at 5′UTR nt 95–110 and NS5B nt 8528–8543 generated by primer-directed PCR mutagenesis (nt positions are based on HCV G1 reference sequence NCBI, NC_004102.1). HCV 5′UTR mutants were generated using pER-1b DNA template, sense (S) primer *hin*dIII-T7HCV(1–19)-S and antisense (AS) primer HCV-5UTR(77–110), Large mutant (LM)-AS or HCV-5UTR(77–110), Small mutant (SM)-AS (Table S1, Integrated DNA Technologies, Skokie, IL, USA) and Platinum Pfx DNA Polymerase (Thermo Fisher Scientific, St. Laurent, QC, Canada). These two 5′UTR mutant DNAs were individually ligated to HCV 5′UTR-core PCR amplicon that was itself generated from HCV-5UTR(111–130)-linker S-primer and HCV core (825–849)-AS primer (Figure S1). After a second amplification of the two ligation products with *hin*dIII-T7HCV(1–19)-S and HCV(825–849)-AS primers, the amplified DNA was cut with *Hin*dIII and *Cla*I restriction enzymes and re-inserted into pER-1b as a cassette to create pER-1b-5′UTR_LM_ and pER-1b-5′UTR_SM_ mutant virus DNA templates. In a similar fashion, NS5B 8528-8543 virus mutants were created as an initial DNA amplification using HCV-NS5B(8060–8084)-Sen and anti-sense primers HCV-NS5B(8511–8546)LM-AS or HCV-NS5B(8511–8546)SM-AS followed by ligation with an HCV NS5B DNA linker sequence created with primers HCV-NS5B(8547–8576)-linker-S and HCV-NS5B(9043–9066)-AS (Figure S1). NS5B primer designs contain changes in the 2nd or 3rd codon nucleotides that are expected to weaken the NS5B-5′UTR RNA duplex but maintain the original RNA polymerase amino acid sequence. The m-fold program [21,22] was used when selecting nucleotides to alter in the 5′UTR(95–110) and NS5B(8528–8543) sequences that would not alter the overall RNA structure when later assembling complementary virus genomes. After a second amplification of the ligation product and with primers HCV-NS5B(8060–8084)-S and HCV-NS5B(9043–9066)-AS, the amplified DNA cassette was cut with *Bgl*II restriction enzyme and re-inserted into pER-1b to create pER-1b-NS5B_LM_ and pER-1b-NS5B_SM_ mutant virus templates. Complementary double HCV mutants, pER-1b-Dbl_LM_ and pER-1b-Dbl_SM_ were created using HCV-5UTR(77–110)LM or HCV-5UTR(77–110)SM cassettes and matching HCV-NS5B(8511–8546)LM-AS or HCV-NS5B(8511–8546)SM-AS cassettes. Predicted HCV1b DNA template pER-1b and mutant virus DNA templates were verified by DNA sequencing.

### 2.3. HCV Production and Immunofluorescence

Infectious virus was produced in Huh-7.5 cells as outlined by Wakita et al 2005 [23]. Genomic HCV RNA was synthesized using the MEGAscript T7 kit (Thermo Fisher Scientific) with 1 µg of *Xba*I-linearized, mung bean nuclease-treated pER-1b or pER-1b containing 5′UTR or NS5B mutations (Thermo Fisher Scientific). RNA transcription reactions proceeded for 4 to 6 h at 37 °C, followed by DNA template removal using RNase-free DNase (Thermo Fisher Scientific), organic extraction and ethanol precipitation. Genomic-length 9.6 kb RNAs were verified for size and homogeneity in 0.8% formaldehyde-agarose gels. HCV RNA (5 μg) was electroporated into 2.5 × 10^6^ Huh-7 cells using a Gene Pulser (BioRad, set at 270 V and 960 μF). Successful electroporation of the HCV genome was verified by detection of HCV antigen immunofluorescence 24 h post-electroporation (Figure S2) [24]. Cells were fixed with methanol, and HCV core and NS5B antigens detected using anti-core and anti-NS5B monoclonal antibodies (TanTec Biosystems, Montreal, QC, Canada), respectively. Bound monoclonal antibodies were detected following incubation with biotin-conjugated goat anti-mouse IgG and dichlorotriazinyl amino fluorescein (DTAF)-conjugated streptavidin (Jackson ImmunoResearch Laboratories, West Grove, PA, USA). Two days post-electroporation, total cellular RNA was extracted with TriReagent (Molecular Research Center, Cincinnati, OH, USA) and the amount of HCV RNA measured by RT-qPCR. Extracellular virus present in the culture medium was measured by infection of freshly seeded Huh-7.5 cells plated at 3 × 10^5^ cells/well in a 96-well plate. Huh-7 cells were inoculated with serial 10-fold dilutions of clarified culture medium (16,000× *g* for 10 min). One day post-infection, total cellular RNA was extracted with TriReagent and HCV RNA was quantified by RT-qPCR. HCV core and NS5B protein expression was assessed by immunofluorescence to determine infectious virus titers as focus-forming units (FFU)/mL [24].

### 2.4. HCV RNA Quantification

The amount of HCV RNA in 1 μg of cellular RNA was performed by RT-qPCR using the HCV sense primer HCV-5UTR(130–146)-S (nt 130–146) and antisense primer HCV-5UTR(272–290)-AS (nt 272–290) (Table S1) and QuantiTect SYBR Green RT-PCR Kit (Qiagen Canada, Montreal, QC, Canada) [25]. Primers were used at final concentrations of 400 nM. HCV cDNA was amplified and quantified using the Mx3000P real-time PCR thermocycler (Thermo Fisher Scientific) for 40 cycles, each consisting of a 15-second incubation at 94 °C, followed by a 30-second annealing and elongation step at 72 °C. Serial concentrations of synthetic HCV RNA genome ranging from 1 pg to 100 ng served as the HCV PCR reference standard.

### 2.5. RNA Binding Assays

Target and probe RNAs were synthesized using the MEGAscript™ T7 kit and DNA templates encoding regions of interest in the 5′UTR and NS5B. DNA templates were generated using pER-1b_WT_ or pER-1b_mutants_ in conjunction with primers *Hin*dIII-T7-HCV(1–19)-S and HCV-5UTR(272–290)-AS or the NS5B 8374–8676 (HCV-NS5B(8374–8392)-S and HCV-NS5B(8657–8676)-AS to yield 5′UTR_(1–290)WT_, 5′UTR_(1–290)LM_ and 5′UTR_(1–290)SM_, NS5B_(8374–8676)WT_, NS5B_(8374–8676)LM_ and NS5B_(8374–8676)SM_ RNAs. RNA probes were uniformly labeled with [α-^32^P]UTP (25 µCi, 800 Ci/mmol, Perkin Elmer, Woodbridge, ON, Canada) and unincorporated isotope was removed using RNA Cleanup of RNeasy Mini Kit (Qiagen Canada).

RNA binding assays were performed as outlined [26]. Briefly, ^32^P-labeled RNA probes were mixed with unlabeled RNAs in 50 mM sodium cacodylate (pH 7.5), 300 mM KCl and 1 mM MgCl_2_, denatured by incubation at 95 °C for 3 min, followed by stepped cooling from 70 °C to 37 °C (1 °C/min). RNA–RNA complexes were allowed to form for 30 min at 37 °C followed by immediate electrophoresis in a 5% native polyacrylamide gel supplemented with 2.5 mM MgCl_2_. Electrophoresis was performed at 4 °C for 1 h at 120 V in TBM buffer (45 mM Tris/HCl (pH 8.3), 43 mM boric acid, 0.1 mM MgCl_2_). Gels were dried and band intensity seen in the autoradiographs was measured using ImageJ software (Rasband WS, National Institutes of Health, Bethesda, MD, USA). K_d_ and B_max_ values were calculated using the nonlinear regression analysis (GraphPad Prism 8.0, San Diego, CA, USA) according to the equation Y = B_max_ × X / (K_d_+X), where X is the concentration of unlabeled RNA, K_d_ is the dissociation constant, Y is the percentage of bound probe and B_max_ is the amplitude of the reaction and represents maximum binding.

### 2.6. RNA Structure Models and Data Analysis

The NS5B RNA secondary structure spanning nt 8490–8560 coding sequence was predicted using mViennaRNA package 2.4.8 [21,27]. RNAcofold [28] was used to predict interactions between the 5′UTR and the NS5B coding sequence [29]. The QuickAlign tool [30] was used in concert with the HCV genotype reference sequences to perform sequence alignment between 5′UTR nt 95–110 and NS5B nt 8528–8543. The average identity match was calculated using ALISTAT [31]. Graphic data were expressed as the arithmetic mean ± SE and the Student’s *t*-test was used when comparing replication differences between wild-type HCV and mutant virus constructs.

## 3. Results

### 3.1. In vitro Complex Formation between 5′UTR_(95–110)_ and NS5B_(8528–8543)_ RNAs

**Figure 1:**
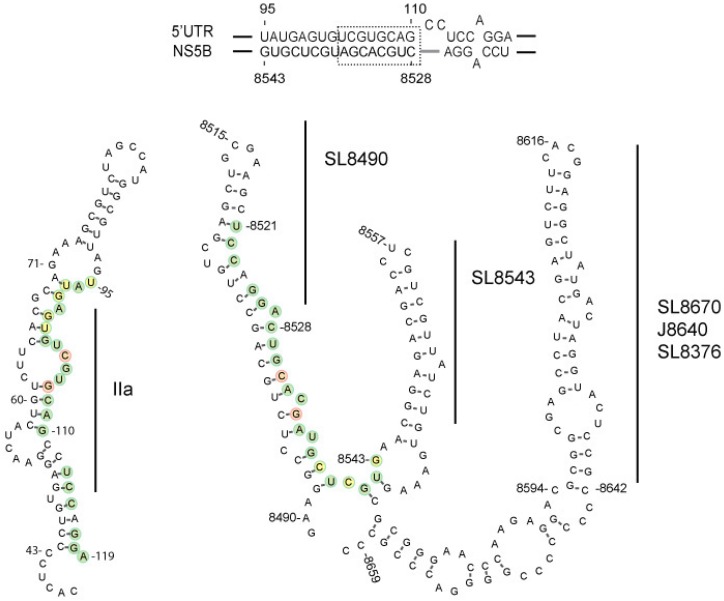
Depiction of minimum free energy duplex model (top panel) and stem-loop structure plots for the IRES domain II (bottom left) and NS5B region 8490–8659 (bottom right). Domain IIa and NS5B sequence pairings (bottom, shaded circles), small mutation SM constructs (red), large mutation LM constructs (red and yellow) and adjacent stem-loop SL8670/J8640/8376 are shown [15].

In silico analysis indicates that a highly conserved (90% 5′UTR and 70% NS5B inter-genotype sequence identity) yet uncharacterized hetero-duplex could be formed by the 5′UTR domain IIa nt 95–110 and NS5B nt 8528–8543, which may contribute to genome circularization and HCV RNA replication (Figure 1) [18]. RNAcofold predicts an optimal minimal free energy (MFE) of -24.0 for a duplex model comprised of HCV IRES nt 95–110 and NS5B nt 8528–8543, and whose interaction is composed of wobble and Watson-Crick base-pairs (Figure 1, hatched boxed). Notably, the base pairing involves domain IIa of the 5′UTR.

**Figure 2:**
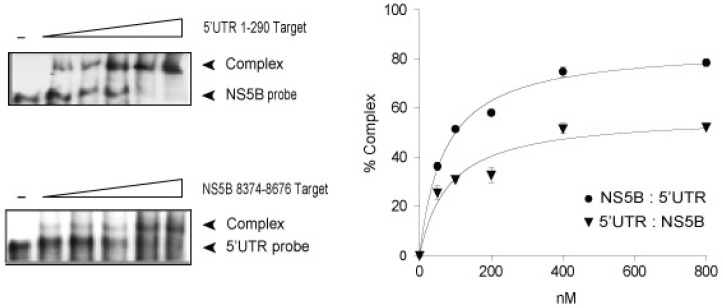
In vitro complex between 5′UTR nt 1–290 and NS5B nt 8374–8676 RNAs. ^32^P-labeled RNA probe (20 nM) was incubated with increased amounts of unlabeled RNA target (50–800 nM) in low magnesium binding buffer. Complexes were separated in a native 5% acrylamide gel buffered with TBM. Plots of % probe complex versus target concentration ± SE were derived from three separate experiments. See Materials and Methods Section 2.5 RNA Binding Assays for derivation of K_d_ and B_max_ values.

An initial investigation for the possible formation of this duplex used in vitro binding assays and HCV 5′UTR nt sequences 1–290 and NS5B nt sequences 8374–8676. These larger sequences, while possessing other SL structures, were chosen for analysis to allow proper contextual folding of the predicted 5′UTR and NS5B pairs [5,32]. Results from RNA:RNA binding experiments in which a fixed amount of ^32^P-labeled NS5B probe was incubated with increased amounts of 5′UTR target RNA showed an average dissociation constant K_d_ value of 70.67 ± 7.20 SE nM (*n* = 3) and an average binding maximum B_max_ value of 85.15 ± 2.25% (Figure 2). Complementary experiments in which ^32^P-labeled 5′UTR probe was incubated with increased concentrations of NS5B target RNA showed comparable K_d_ and B_max_ values (83.93 nM ± 21.97 SE nM and 57.32 ± 4.01%, respectively) (Figure 2). These K_d_ and B_max_ values are consistent with other reported high-affinity interactions between the HCV IRES IIId and NS5B SL 3.2 [33].

**Figure 3:**
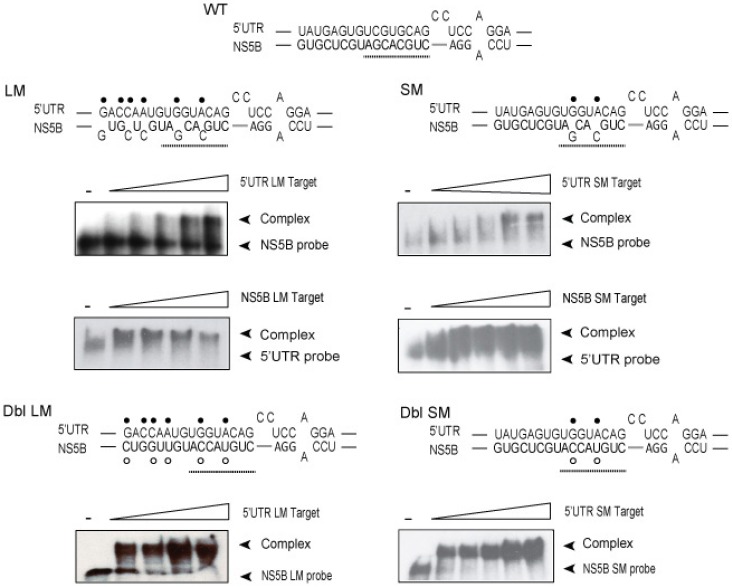
In vitro complex between 5′UTR nt 1–290 and NS5B nt 8374-8676 RNAs containing six and five mutations (LM) or two mutations (SM) in the 5′UTR nt 95–110 (•) or NS5B nt 8528-8543 (◦). ^32^P-labeled RNA probe (20 nM) was incubated with two-fold increasing amounts of unlabeled RNA target (six lane gels = 0, 50, 100, 200, 400, 1000 nM) or (five lane gels = 0, 50, 100, 200, 400 nM) in low magnesium binding buffer. Plots of % probe complex versus target concentration ± SE were derived from three separate experiments and used to calculate K_d_ and B_max_ values as summarized in Table 1.

In order to determine which nucleotides within the two RNA sequences were required to form the stable duplex, we constructed RNA molecules spanning the entire 5′UTR nt 1–290 and NS5B nt 8374–8676 complex. RNA construct designated as 5′UTR(1–290) large mutant (LM) contained six altered nucleotides in 5′UTR nt 95–110 (Figure 3, •, and Figure 1, red and yellow circles). RNA construct designated as NS5B(8374–8676)LM contained five altered nucleotides in NS5B nt 8528-8543 (Figure 3A, ◦, and Figure 1 yellow and red circles). These two constructs were expected to disrupt predicted wobble and Watson-Crick base-pairings and spanned the entire duplex. Two other RNA constructs designated as 5′UTR(1–290) small mutant (SM) and NS5B(8374–8676)SM, each containing two altered nucleotides, were expected to disrupt predicted Watson-Crick base-pairing (Figure 3, SM, ◦ and •, and Figure 1 red circles). Altered nucleotides were selected to be silent mutations in the RNA-dependent RNA polymerase coding sequence and to reconstitute a novel complementary duplex when matching RNAs were present (Figure 3, Dbl).

Results from RNA:RNA binding experiments using ^32^P-labeled NS5B or 5′UTR in combination with large and small mutant target RNAs showed a 5.2- and 6.6-fold increase in K_d_ values for 5′UTR(95–110) and NS5B(8528–8543) large mutations as compared to WT HCV RNA (Figure 3 and Table 1). Small mutations in 5′UTR(95–110) and NS5B(8528–8543) showed a 4.5- and 5.8-fold increase in K_d_ values as compared to WT HCV RNA (Figure 3 and Table 1). Overall, these findings indicate that mutations predicted to lessen WT duplex MFE (i.e., −24) greatly decreased duplex affinity (Table 1). The results also revealed that the presence of just two Watson-Crick base pairings in the NS5B nt8528–8543 sequence has a larger negative impact (5.8-fold) on duplex affinity as compared to their 5′UTR counterpart (4.5-fold). In contrast, large and small complementary mutations for the 5′UTR and NS5B showed K_d_ values comparable to those found for WT HCV RNA (Table 1, NS5B-LM:5′UTR-LM, 110% and NS5B-SM:5′UTR-SM, 130%, respectively) and suggests that compensatory mutations are able to rescue base-pair affinity. B_max_ values were most impacted by mutations in the 5′UTR (Table 1). Both the 5′UTR(95–110)LM and 5′UTR(95–110)SM showed a 42% decrease, whereas mutations in the NS5B(8528–8543) showed B_max_ values ≤ 24% (Table 1). B_max_ values for large and small complementary mutations were also comparable to WT HCV RNA (Table 1, NS5B-LM:5′UTR-LM, 98% and NS5B-SM:5′UTR-SM, 118%, respectively). The 42% decrease in B_max_ values when using 5′UTR-LM or 5′UTR-SM as targets indicates that a mutation in 5′UTR IIa has a larger negative impact on the rate of complex formation.

These results demonstrate that mutations in one of the two sequences predicted to be involved in the interaction of 5′UTR_(1–290)_ and NS5B_(8374–8676)_ RNAs cause a loss of binding affinity. Moreover, binding assays realized with molecules containing compensatory mutations were able to reconstitute duplex interaction.

### 3.2. Disruption of the Distal Interaction between 5′UTR nt 95–110 and NS5B nt 8528–8543 Decreases HCV RNA and Virus Production

In vitro binding assays showed decreased duplex affinity and lesser rates of complex formation when small changes were introduced into 5′UTR or NS5B sequences. To determine whether the changes observed in vitro would impact virus replication, we constructed HCV genomes containing 5′UTR_LM_, 5′UTR_SM_, NS5B_LM_, NS5B_SM_, as well as virus genomes containing the large and small complementary mutations, called Double-LM (Dbl_LM_) and Double-SM (Dbl_SM_). The former four mutant genome constructs were expected to reduce the formation or stability of the 5′UTR:NS5B complex while the latter two genome constructs (Dbl_LM_ and Dbl_SM_) were expected to reconstitute the 5′UTR and NS5B complementary annealing, restore complex formation or stability, and restore virus replication. To ensure that any observed changes in virus replication were not due to altered viral RNA-dependent RNA polymerase amino acid sequences, our constructs contain only silent mutations in the second or third codon nucleotides, which were expected to weaken NS5B-5′UTR RNA duplex free energy while maintaining the native RNA polymerase amino acid sequence.

**Figure 4:**
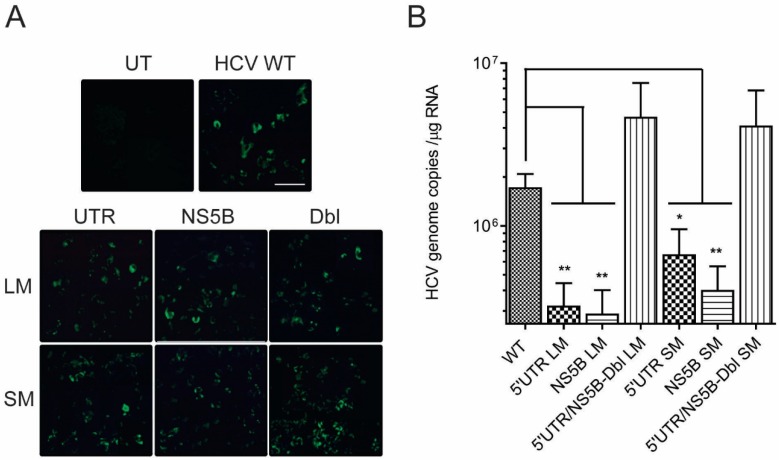
Disruption in the 5′UTR nt95–110:NS5B nt8528–8543 duplex reduces intracellular HCV RNA levels. (**A**) Huh-7.5 cells transfected with HCV genomic RNA (WT) or HCV genomic RNA containing mutations at 5′UTR nt 95–110 and NS5B nt 8528–8543 express NS5B antigen 24 h post-transfection. Scale bar = 50 µm. (**B**) Plots represent the average number of HCV RNA genome copies ± SE in 1 μg of cellular RNA at 48 h post-transfection. *p*-values ≤ 0.05 (*) or ≤ 0.01 (**) were determined by the Student’s *t*-test and represent four or more independent experiments.

Twenty-four hours post-electroporation Huh-7.5 cells showed qualitatively positive NS5B antigen expression (Figure 4A), indicating that our genome constructs were capable of HCV polyprotein synthesis. At 48 h, a time noted for the commencement of HCV RNA synthesis [34], RT-qPCR data indicated that HCV genome constructs containing 5′UTR_LM_, NS5B_LM_, 5′UTR_SM_ and NS5B_SM_ produced on average 5.3- (*p* = 0.006), 6- (*p* = 0.005), 2.6- (*p* = 0.03) and 4.2-fold (*p* = 0.008) less RNA as compared to parental genome (Figure 4B and Table 2). HCV RNA production in Huh-7.5 cells transfected with complementary mutants Dbl_LM_, or Dbl_SM,_ restored RNA levels to those of the parental genome RNA (1.9-fold, *p* = 0.4 and 1.7-fold, *p* = 0.2) (Figure 4B and Table 2).

**Figure 5:**
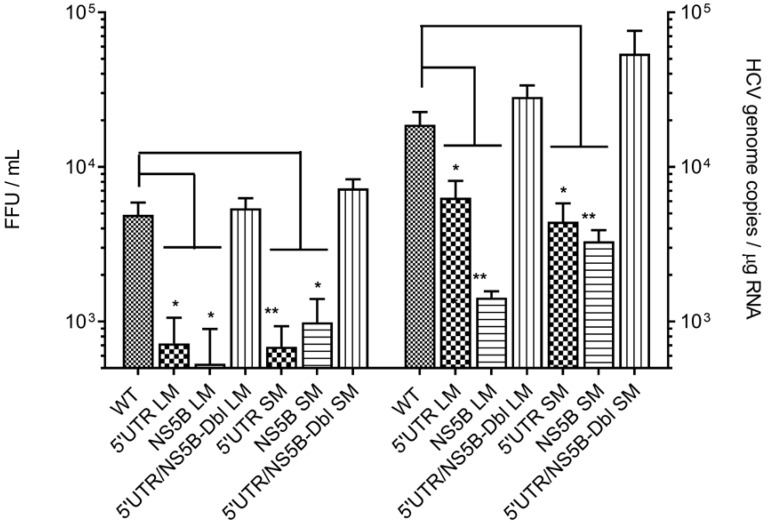
Disruption in the 5′UTR nt 95–110:NS5B nt 8528–8543 duplex reduced progeny virus titers. Huh-7.5 cells were infected with HCV parental virus (WT) or HCV containing mutations at 5′UTR nt 95–110 and NS5B nt 8528–8543. Plots represent the average number of infectious virus titers as focus-forming units (FFU)/mL or HCV RNA genome copies ± SE in 1 μg of cellular RNA taken at 48 h post-infection. *p*-values ≤ 0.05 (*) or ≤ 0.01 (**) were determined by the Student’s *t*-test and represent six independent experiments.

Progeny virions and their subsequent cellular RNA levels were also lower for genome constructs containing mutations in 5′UTR nt 95–110 and NS5B nt 8528–8543. Progeny virus derived from genome constructs containing 5′UTR_LM_, NS5B_LM_, 5′UTR_SM_ or NS5B_SM_ produced on average 4.8- (*p* = 0.02), 3.2- (*p* = 0.04), 10- (*p* = 0.009) and 7-fold (*p* = 0.01) lower virus titers compared to parental genome (Figure 5 and Table 2). Progeny virus produced in Huh-7.5 cells freshly infected with complementary mutant virus, Dbl_LM_ or Dbl_SM_, restored virus titers to levels observed with parental genome RNA (1.37-fold, *p* = 0.4 and 1.7-fold, *p* = 0.2) (Figure 5 and Table 2).

Progeny virus cellular RNA levels from genome constructs containing 5′UTR_LM_, NS5B_LM_, 5′UTR_SM_ or NS5B_SM_ produced on average 3- (*p* = 0.01), 13- (*p* = 0.006), 4- (*p* = 0.01) and 6-fold (*p* = 0.001) less virus RNA copies compared to parental genome (Figure 5 and Table 2). Progeny virions containing complementary mutant virus, Dbl_LM_, or Dbl_SM_, restored HCV RNA genome copy levels to those seen with parental genome RNA (1.7-fold, *p* = 0.1 and 1.3-fold, *p* = 0.06) (Figure 5 and Table 2).

Taken together, these results show that the long-range interaction between the 5′UTR nt 95–110 and NS5B nt 8528–8543 is necessary for efficient virus production. Disruptions of the 5′UTR at nt 107 and nt 104, or of NS5B at nt 8531 and nt 8534 are able to significantly lower HCV RNA production and virus titers (Figure 5), and their negative effects can be reversed through complementary pairing. This latter observation suggests that it was functional annealing and restoration of RNA stem structure, and not the requirement for a specific nucleotide sequence, which restored virus replication.

## 4. Discussion

**Figure 6:**
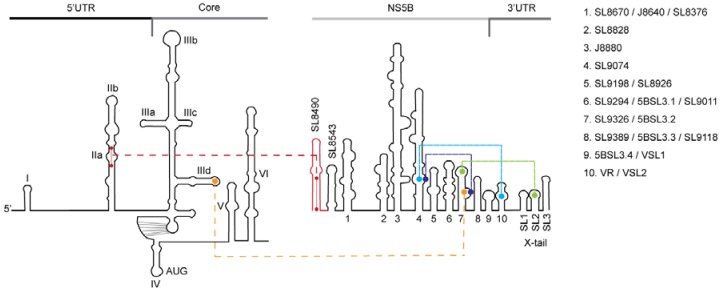
Secondary structural elements located in the 5′UTR-Core and the NS5B-3′UTR. Tertiary interactions between 5′UTR IIa nt 95–110 and NS5B SL8490 nt 8528–8543 are indicated by the red line. Tertiary interactions of SL9326 with the 3′UTR X-tail SL2 are indicated by the green line. SL9326 interaction with NS5B SL9074 and the 3′UTR Variable Region (VR, VSL2) is indicated by the blue and turquoise lines, respectively. SL9326 interaction with the IRES IIId apical loop is indicated by the yellow line. Listed NS5B-3′UTR stem-loop nomenclature is based on Adams RL et al. 2017, Figure 1 [15].

Flavivirus genome secondary and tertiary RNA conformations and their distal interactions serve as a balance between the two major viral events of RNA translation and RNA replication [35,36,37,38]. However, there is limited knowledge of the dynamics of these conformational changes, as well as their orchestration and coordination with viral and cellular replication cofactors. Growing evidence obtained from studies on other members of the *Flaviviridae* supports the importance of RNA secondary structure contact between the distal 5′- and 3′-ends of the HCV genome in the control of virus replication [13,15,39]. It is well documented that the SL rich region in the NS5B coding sequence controls both RNA replication [40] and RNA genome translation (Figure 6) [41]. SL9326 (synonymous with NS5BSL3.2) acts as the central hub through its apical kissing-loop interaction with the 3′UTR X-tail SL2 to control RNA (−)strand synthesis (Figure 6, green line) [40,42]. The stem bulge of SL9326 alternates duplex formation with the NS5B SL9074 (Figure 6, blue line) [43,44], the 3′UTR variable region (VR, VSL2) segment 9387-ACACUCCAGGCC-9398 (Figure 6, turquoise line) [45] and the IRES IIId (Figure 6, yellow line) [41]. This latter interaction serves as a negative regulator of IRES-driven translation.

We observed that 5′UTR nt 1–290 and NS5B nt 8374–8676 RNA sequences formed a high-affinity complex at low magnesium concentrations and in the absence of cell proteins which was controlled largely by HCV domain IIa nt 95–110 and NS5B nt 8528–8543 sequences (Figure 2 and Figure 3). 5′UTR nt 95–110 and NS5B nt 8528–8543 form a stem duplex composed of canonical Watson-Crick and wobble base-pairing [46]. LM constructs that contained six and five alterations in 5′UTR nt 95–110 or NS5B nt 8528–8543 sequences, respectively, and predicted to decrease MFE 3-fold for a predicted duplex, were shown to reduce complex affinity 6-fold (Kd_LM_/Kd_WT_, Table 1, rows 1 and 3). SM mutations (two nucleotides) within the 5′UTR nt 95–110 or NS5B nt 8528–8543 and predicted to decrease MFE 1.6-fold reduced complex affinity 5-fold (Kd_LM_/Kd_WT_, Table 1, rows 2 and 4). In contrast, large and small complementary mutations at these same mutation sites were able to rescue base-pairing and restore affinity (K_d_) values to those of unaltered HCV RNA (Kd_LM_/Kd_WT_, Table 1, rows 5 and 6). An examination of these same mutations within the context of the full-length HCV genome and their participation in virus replication showed that interaction between 5′UTR nt 95–110 and NS5B nt 8528–8543 was necessary for maximum virus RNA levels and progeny virus production (Figure 4 and Figure 5). As outlined in Table 2, LM constructs within these two HCV genome locations reduced intracellular HCV RNA and progeny virus 5.6-fold and 4-fold, respectively. SM constructs within these two HCV genome locations reduced intracellular HCV RNA and progeny virus 3.4-fold and 8.5-fold, respectively (Table 2). The cell culture assays revealed that reconstitution of 5′UTR nt 95–110:NS5B 8528–8543 duplex structure restored both intracellular RNA and virus progeny to levels comparable to unaltered HCV genome RNA (Figure 5). As these complementary mutations differed in composition compared to the parental genome, the restoration of intracellular RNA levels and virus titers suggests that the RNA structure formed by these two distal sequences, and not specific base-pairs, is the overriding mechanism for efficient HCV replication.

Structural studies of the HCV IRES domain II indicates a large extended SL structure [47] that allows the uncapped HCV coding sequence translation-access with the 40S ribosomal subunit [32]. An RNA flexible bulge motif within domain IIa and juxtaposed to 5′UTR nt 95–110 [48] is thought to act as a translation control switch [49,50], but might also serve to moderate RNA replication [48]. Mutations in the NS5B SL J8640, which is next to NS5B 8528–8543, were shown to reduce intracellular RNA levels and progeny virus 2- and 20-fold, respectively [16]. Our findings of reduced RNA levels and progeny virus titers for LM and SM genome constructs (Table 2) are in agreement with these earlier observations and support the idea that preservation of 5′UTR nt 95–110 and NS5B nt 8528–8543 duplex structural integrity acting in concert with adjacent SL structures is essential for efficient virus replication (Figure 1). From our observed interaction of NS5B nt 8528–8543 with IRES domain IIa (Figure 6, red line), we speculate that NS5B nt 8528–8543 acts in concert with SL9326 (5BSL3.2) to repress IRES binding to the 40S ribosomal subunit [41] and formation of the 80S ribosomal complex [51]. Ultimately, NS5B’s disruption of IRES structures and viral protein synthesis in concert with NS5B’s repositioning of the 3′UTR would switch the HCV genome from a translation template to a replication template.

## 5. Conclusions

Long-range interaction between RNA duplexes located at the distal ends of the HCV genome are hypothesized to facilitate and regulate important stages in the virus replication cycle. The data presented here indicate that a novel hetero-duplex formed by 5′UTR nt 95–110 and NS5B nt 8528–8543 is essential for optimal virus RNA production. Virions containing silent mutations within these two sequences, and expected to disrupt duplex annealing, were shown to decrease both cellular HCV RNA levels and progeny virus titers when compared to the parental virus. Virions containing complementary silent mutations within 5′UTR nt 95–110 and NS5B nt 8528–8543, and expected to restore RNA annealing, were shown to increase virus RNA and progeny virus titers to levels comparable to the parental virus.

## Figures and Tables

**Table 1:** Binding variables of the 5′UTR–NS5B RNA interaction.

RNA Probe	Target RNA	Avg K_d_ + SD (nM) ^1^	B_max_ (%) ^1^	K_dMT_/K_dWT_	% B_max_ MT/WT	MFE
NS5B-WT	5′UTR-LM	364.7 ± 124.6	49.78 ± 7.47	5.2	58.4	−6.9
NS5B-WT	5′UTR-SM	314.5 ± 120.8	49.64 ± 7.55	4.5	58.3	−13.9
5′UTR-WT	NS5B-LM	551.8 ± 151.1	54.77 ± 6.40	6.6	95.6	−6.9
5′UTR-WT	NS5B-SM	489.8 ± 129.4	43.77 ± 5.14	5.8	76.4	−14.2
NS5B-LM	5′UTR-LM	74.84 ± 7.28	83.35 ± 2.15	1.1	97.8	−24.6
NS5B-SM	5′UTR-SM	72.35 ± 8.04	67.45 ± 1.85	1.3	118	−22.6

^1^ N = three independent experiments; K_d_, dissociation constant; B_max_, maximum binding amplitude; MFE, minimal free energy.

**Table 2:** HCV RNA levels and progeny virus titers in Huh-7 cells transfected with 5′UTR and NS5B mutant genome constructs.

Virus Construct	HCV RNA Copies ^1^	MT WT	Progeny Virus ^1^	MT WT	Progeny HCV RNA Copies ^2^	MT WT
WT genome	1.7 × 10^6^ ± 3.5 × 10^5^		6.9 × 10^3^ ± 2.1 × 10^3^		1.9 × 10^4^ ± 3.8 × 10^3^	
5′UTR_LM_	3.2 × 10^5^ ± 1.2 × 10^5^	−5.3	1.4 × 10^3^ ± 7.6 × 10^2^	−4.8	6.3 × 10^3^ ± 1.8 × 10^3^	−2.9
NS5B_LM_	2.9 × 10^5^ ± 1.1 × 10^5^	−6.0	2.1 × 10^3^ ± 1.0 × 10^3^	−3.2	1.4 × 10^3^ ± 1.4 × 10^2^	−13.1
5′UTR_SM_	6.7 × 10^5^ ± 2.9 × 10^5^	−2.6	6.9 × 10^2^ ± 2.4 × 10^2^	−10	4.4 × 10^3^ ± 1.3 × 10^3^	−4.3
NS5B_SM_	4.0 × 10^5^ ± 1.6 × 10^5^	−4.2	9.9 × 10^2^ ± 4.0 × 10^2^	−7	3.3 × 10^3^ ± 5.9 × 10^2^	−5.7
Dbl_LM_	4.7 × 10^6^ ± 2.9 × 10^6^	1.9	7.2 × 10^3^ ± 1.9 × 10^3^	2	2.8 × 10^4^ ± 5.3 × 10^3^	1.7
Dbl_SM_	4.1 × 10^6^ ± 2.7 × 10^6^	1.7	9.4 × 10^3^ ± 2.3 × 10^4^	1.7	5.4 × 10^4^ ± 2.2 × 10^4^	1.3

^1^ Focus-forming units (FFU)/mL ± SE; ^2^ HCV genome copies/1 μg total RNA ± SE.

## Supplementary Materials

The following are available online at http://www.mdpi.com/1999-4915/11/1/17/s1, Figure S1: Map of HCV genome showing the HindIII-5′UTR-Core-ClaI and BglII-NS5B cloning cassettes, Figure S2: HCV cell culture system, Table S1: Primers used in HCV mutant construction and qPCR assays.
