# Supplementary material for: Genomic-Scale Interaction Involving Complementary Sequences in the Hepatitis C Virus 5′UTR Domain IIa and the RNA-Dependent RNA Polymerase Coding Region Promotes Efficient Virus Replication
Source: Viruses. 2018 Dec 28;11(1):17. doi: 10.3390/v11010017 (PMC6357077; doi:10.3390/v11010017)
Supplement: Supplementary file 1 [file viruses-11-00017-s001.pdf]

Table S1. Primers used in HCV mutant construction and qPCR assays

Primer[1]	Sequence[2]
***Hin*dIII**-T7-5′UTR:1-19S	**AAGCTTG**TAATACGACTCACTATAGCCAGCCCCCGATTGGGGG
5′UTR:77-110LM AS	CTGTCCACATTGGTCCTAACGCCATGGCTAGAC
5′UTR: 77-110SM AS	CTGTACCACACTCATACTAACGCCATGGCTAGAC
5′UTR: 111-130 Linker S	CCTCCAGGACCCCCCCTCCC
HCV Core: 825-849 AS	GCAGATTCCCTGTTGCATAGTTCAC
NS5B 8060-8084 S	TAAGCCAGCCCGCCTTATCGTATTC
NS5B 8511-8546 BM AS	GTTGACCAACATGGTACAGTCCTGGAGCTTCGCAGC
NS5B 8511-8546 SM AS	GTTCACGAGCATGGTACAGTCCTGGAGCTTCGCAGC
HCV NS5b(8547-8576) linker S	GGAGACGACCTCGTCGTTATCTGTGAAAGC
NS5B 9043-9066 AS	TGAGGCATGAAGCCACCCTATTGA
HCV 5′URT (130-146) S	CGGGAGAGCCATAGTGG
HCVUTR 272-290) AS	AGTACCACAAGGCCTTTCG
HCV NS5B (8374-8392) S	TAATACGACTCACTATAGGGCTTTATATCGGGGGTCC
Hcv ns5b (8657-8676) AS	AGTCGTATTCTGGTTGGGGC

[1] S, Sense; AS, Antisense.

[2] **AAGCTTG**, *Hin*dIII recognition size; **G**TAATACGACTCACTATAG, T7 RNA polymerase promoter; N, mutated nucleotide.

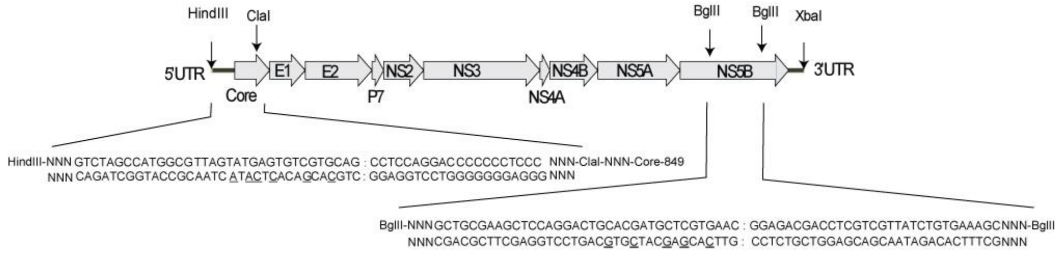

Figure S1. Map of HCV genome showing the *Hin*d III-5′UTR-Core-*Cla*I and *Bgl*II-NS5B cloning cassettes. The nucleotides changed (N) within IRES domain II nt 95-110 and NS5B nt 8528 to 8543 and the 5′ sequence of the blunt ligation partner (:) are shown and as outlined in Materials and methods, *Section 2.2 Construction of HCV 1b mutant virus.*

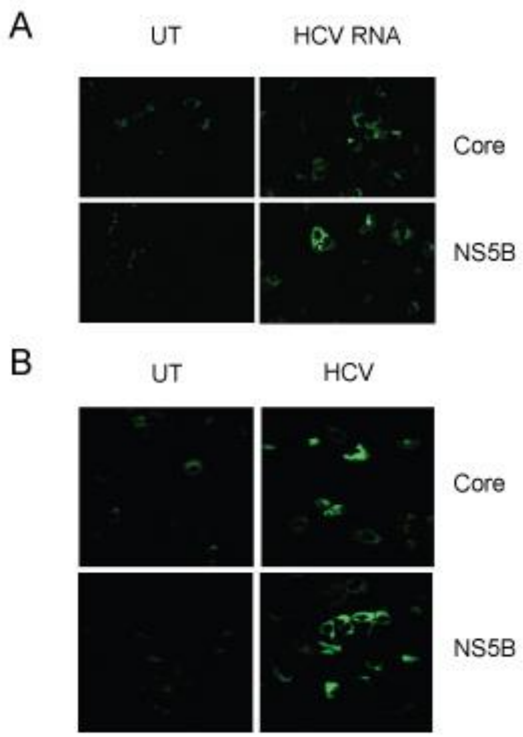

Figure S2. HCV cell culture system. Huh-7.5 cells were transfected or infected with full-length HCV RNA or HCV, respectively. (A) Immunofluorescence expression of HCV core antigen (top row) or NS5B antigen (bottom row) in mock-transfected (UT) or HCV 1b RNA-transfected Huh-7.5 cells as measured at 2 days post-transfection. (B) Immunofluorescence expression of HCV core antigen (top row) or NS5B antigen (bottom row) in mock-infected (UT) or HCV-infected Huh 7.5 cells as measured at 2 days post-infection.
